# The first steps in the evaluation of a "black-box" decision support tool: a protocol and feasibility study for the evaluation of Watson for Oncology

**Published:** 2018-07-27

**Authors:** Lotte Keikes, Stephanie Medlock, Daniel J. van de Berg, Shuxin Zhang, Onno R. Guicherit, Cornelis J.A. Punt, Martijn G.H. van Oijen

**Affiliations:** ^1^Department of medical oncology, Cancer Center Amsterdam, Academic Medical Center, University of Amsterdam, the Netherlands; ^2^Department of medical informatics, Academic Medical Center, University of Amsterdam, Amsterdam, the Netherlands; ^3^Department of Surgery, University Cancer Center Leiden/the Hague, the Hague, the Netherlands

**Keywords:** evidence-based care, decision support tool, Watson for Oncology, clinical practice guideline, quality of care, personalized medicine

## Abstract

**Background and aim:**

Medical specialists aim to provide evidence-based care based on the most recent scientific insights, but with the ongoing expansion of medical literature it seems unfeasible to remain updated. "Black-box" decision support tools such as Watson for Oncology (Watson) are gaining attention as they offer a promising opportunity to conquer this challenging issue, but it is not known if the advice given is congruent with guidelines or clinically valid in other settings. We present a protocol for the content evaluation of black-box decision support tools and a feasibility study to test the content and usability of Watson using this protocol.

**Methods:**

The protocol consists of developing synthetic patient cases based on Dutch guidelines and expert opinion, entering the synthetic cases into Watson and Oncoguide, noting the response of each system and evaluating the result using a cross-tabulation scoring system resulting in a score range of —12 to +12. Treatment options that were not recommended according to the Dutch guideline were labeled with a "red flag" if Watson recommended it, and an "orange flag" if Watson suggested it for consideration. To test the feasibility of applying the protocol, we developed synthetic patient cases for the adjuvant treatment of stage I to stage III colon cancer based on relevant patient, clinical and tumor characteristics and followed our protocol. Additionally, for the feasibility study we also compared the recommendations from the NCCN guideline with Watson's advice, and evaluated usability by a cognitive walkthrough method.

**Results:**

In total, we developed 190 synthetic patient cases (stage I: n=8; stage II: n=110; and stage III: n=72). Overall concordance scores per case for Watson versus Oncoguide ranged from a minimum score of -4 (n=6) to a maximum score of+12 (n=17) and from —4 (n=9) to +12 (n=24) for Watson versus the NCCN guidelines). In total, 69 cases (36%) were labeled with red flags, 96 cases (51%) with orange flags and 25 cases (13%) without flags. For the comparison of Watson with the NCCN guidelines, no red or orange flags were identified.

**Conclusions:**

We developed a research protocol for the evaluation of a black-box decision support tool, which proved useful and usable in testing the content and usability of Watson. Overall concordance scores ranged considerably between synthetic cases for both comparisons between Watson versus Oncoguide and Watson versus NCCN. Non-concordance is partially attributable to guideline differences between the United States and The Netherlands. This implies that further adjustments and localization are required before implementation of Watson outside the United States.

**Relevance for patients:**

This study describes the first steps of content evaluation of a decision support tool before implementation in daily oncological patient care. The ultimate goal of the incorporation of decision support tools in daily practice is to improve personalized medicine and quality of care.

## 1 Introduction

Medical specialists do their best to provide high-quality, evidence-based care based on the latest scientific insights, but it is very difficult to keep up with the increasing amount of med¬ical literature in combination with the time demands of daily pa¬tient care. Computerized clinical decision support systems can help address this challenging issue, if the system is able to judge and summarize available medical literature and generate person¬alized treatment advice based on scientific evidence. Digital decision support tools include both simple algorithms such as flowcharts and decision trees, and more complex systems that use artificial intelligence to provide personalized treatment ad¬vice. The latter are considered best for improving clinician per¬formance, but these tools should be evaluated before being im¬plemented in routine daily practice, and usability of the systems remains variable [1].

In the last decade, several "artificial intelligence", "machine learning", or "cognitive computing" medical decision support initiatives have gained attention. One of these tools is IBM Watson for Oncology (abbreviated as Watson) [2-4]. Watson uses natural language processing to extract data from free text in medical records and select treatments from consensus guidelines [5]. Its selection of treatments is refined using machine learning, trained by specialists from New York's Memorial Sloan Ketter¬ing Cancer Center [5]. This combination of technologies has the potential to solve two major problems in the field of decision support: harnessing data from poorly-structured medical record data and keeping the medical knowledge base of the system up-to-date [6]. Clinicians are used to recording patient data in nat¬ural language. However, most decision support systems require structured data (e.g. coded diagnoses) to function properly. Wat¬son has the potential to circumvent this problem by using natural language processing to interpret the unstructured data in the pa¬tient record. Similarly, most decision support systems rely on a knowledge base that is constructed by manual translation of one or more clinical guidelines into software. Guidelines must also be updated by hand, which is time-consuming (i.e. months to years). By automatically integrating evidence [7], Watson has the potential to offer advice that is more up-to-date, as well as of¬fer more personalized advice through case-based reasoning [5].

In theory, Watson's approach of integrating natural language processing with evidence from guidelines and studies, case-based reasoning, and machine learning based on training by ex¬pert oncologists could result in better advice than simply follow¬ing a guideline, but Watson is a "black- box" system - its rea¬soning is opaque to the user. As long as the underlying internal processes and technology that explain Watson's way of operating are not publicly available, the process of reasoning cannot be ex¬ternally evaluated. Even if the complete system were available for external review, it is difficult or impossible to infer how a neural-network-based system such as Watson uses the available data to reach its conclusions [4]. This brings unique challenges in evaluating software like Watson prior to clinical use.

Evaluation of software is often divided into two steps: veri-fication and validation [8]. Verification is checking whether the system was built according to specification. In a typical rule-based decision support system, this would involve testing the individual rules to ensure that the system provides the expected output for a given set of inputs. The second step is validation or checking that the system meets user expectations. In a typical decision support system, this would involve giving set of cases (selected based on the range of expected inputs and outputs) to clinicians and asking them to compare the output of the system to their own assessment. This testing should be done before con¬ducting a clinical trial, which is aimed to assess the impact of the system on actual clinical decision-making [9].

However, in a black-box system like Watson, this approach is not possible. The exact inputs (the data that the system uses in its reasoning) and expected outputs are opaque to the user. Previous efforts in evaluating Watson have thus far only been reported as conference abstracts [10-12], and these short reports indicate that the evaluations consisted of comparing the output of Watson in actual clinical cases against the evaluation of clin¬ical experts. However, although this approach approximates the validation step of a typical evaluation, a selection of consecu¬tive clinical cases probably represents only a small sample of possible cases. Common cases are likely to be overrepresented. Unusual cases may not appear at all. Since machine learning systems tend to perform better when they have been trained with more data, Watson may also perform better in common cases than in unusual ones - and since it is precisely these unusual cases where clinicians may seek advice, a systematic approach to testing is needed before performing an impact study. Smith *et al. *suggested a general approach to evaluation of such systems, which involves comparing the performance of such systems to a validated gold standard [13]. However, as is often the case in medicine, no gold standard exists in oncology. Furthermore, Watson's use of free text data complicates the analysis by intro¬ducing uncertainty about the spectrum of cases, which should be tested.

Watson is already supporting cancer care in more than 150 hospitals in 11 countries as published on IBM's website [14]. It is unclear if Watson's system is localized to the cases and clin-icians on which the system was trained. The aim of our study is therefore to present a protocol for the systematic evaluation of "black-box" decision support tools in general and, to demon¬strate the feasibility of this approach with an evaluation of Wat¬son's performance in a specific clinical situation - choice of ad¬juvant therapy for colorectal cancer.

The aim of our study is therefore to present a protocol for the systematic evaluation of "black-box" decision support tools in general and, to demonstrate the feasibility of this approach with an evaluation of Watson's performance in a specific clini¬cal situation - choice of adjuvant therapy for colorectal cancer.

**Figure 1.**
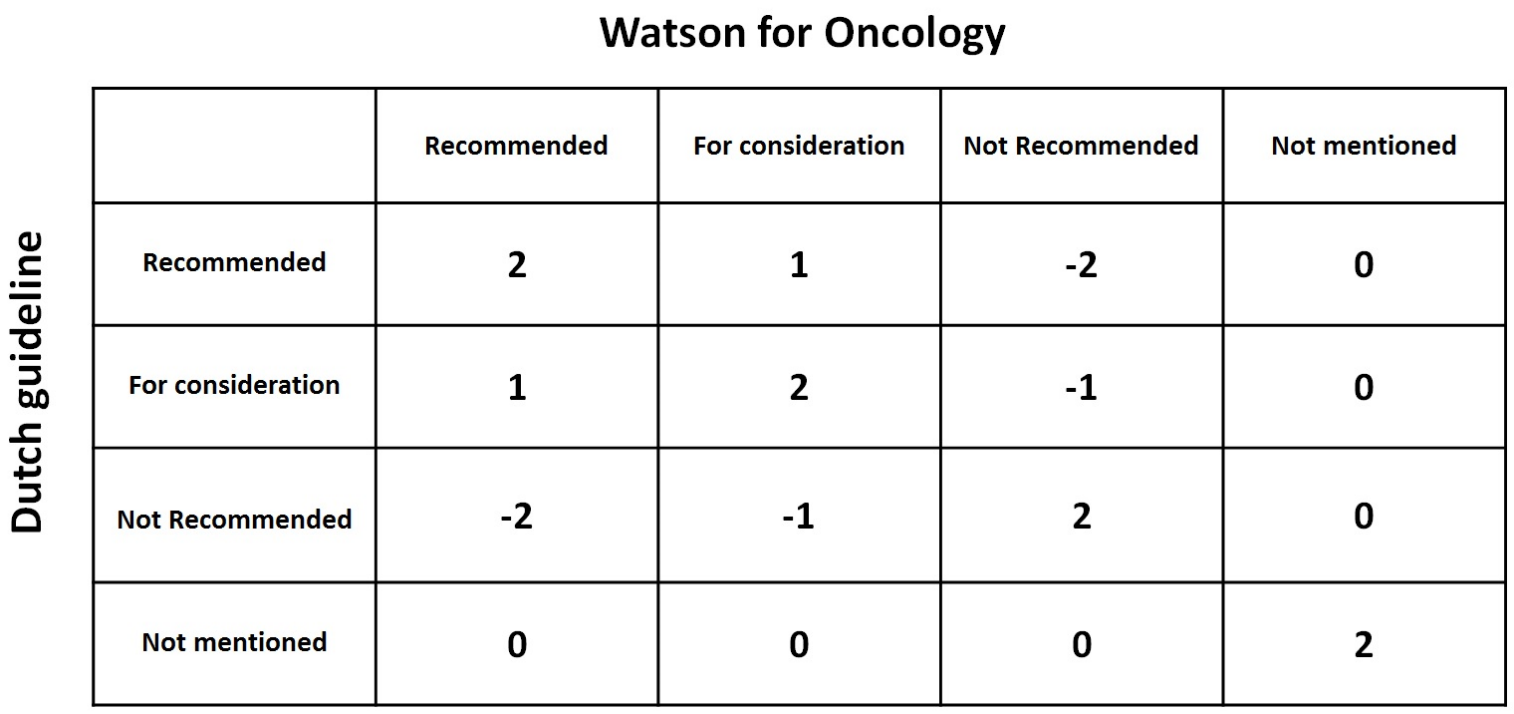
Matrix with levels of concordance comparing the Dutch guideline and Watson for Oncology recommendations

## 2 Methods

### 2.1. Protocol

Content evaluation. Patient cases with known medical treatment decisions or synthetic patient cases with corres¬ponding guideline recommendations could be used to test the concordance of a decision support tool with the Dutch national clinical guidelines.

#### 2.1.1. Generating synthetic patient cases

We plan to evaluate Watson for colorectal cancer by com¬paring it with the Dutch colorectal cancer guideline. Decision trees representing this guideline are available as open access software (available at www.oncoguide.nl). As Watson's reason-ing is opaque to the user, we will rely on empirical testing. We will generate synthetic patient cases instead of using real patient data to exhaustively test for differences with the guideline rec¬ommendations. Synthetic patients are generated to test all paths through the guideline, which result in a different recommenda-tion. Each patient, clinical or tumor characteristic, which could lead to a different recommendation, is considered a 'decision point'. Decision points in the Dutch guideline are extracted from the decision trees in the Oncoguide software [15], including characteristics which can influence clinical decisions in practice but are not mentioned in the Dutch guideline, as defined by clini¬cal experts. For categorical variables, we will test each value. For continuous variables such as age, we will select a purposive sample of values (e.g. we select 3 ages representing relatively young, average, and older patients). The newest versions of both systems will be used.

#### 2.1.2. Processing Watson's advice

For each case, we will enter a minimum set of variables into an interface created by MRDM (Medical Research Data Management) to generate Watson's treatment advice per case. Treatment options from Watson are given in categories of recom-mended (R), for consideration (C) and not recommended (NR), with supporting medical literature. Watson's treatment recommendations and background information are saved for each case for analysis.

#### 2.1.3. Comparison to Dutch guidelines

Concordance of a decision support system is not adequately measured by binary agreement or disagreement [8]. Therefore, concordance on treatment per case between Watson's advice and the Dutch guideline recommendations will be evaluated using cross tabulations (Table 1), extending the method suggested by Friedman and Wyatt [8]. The first column lists all potential treat¬ment options in the adjuvant setting of colon cancer and the sec¬ond and third columns are used to enter treatment advice from the Dutch guideline (using Oncoguide software) (column 2) and Watson (column 3). Any comments may be entered in the last column. The treatment advice is then analyzed using a scoring system with a range from -2 to +2, as presented in(Table 2)and furtherexplainedinTable3. Aconcordancematrixispresented in Figure 1. The treatment scores are summed to form an over-all concordance score for each case. Cases are labeled with a 'flag' if Watson considers (orange flag) or recommends (red flag) chemotherapy that is not indicated according to the Dutch guide¬line. If a case has recommendations that qualify it for both red and orange flags, it is labeled with only a red flag.

**Figure 2.**
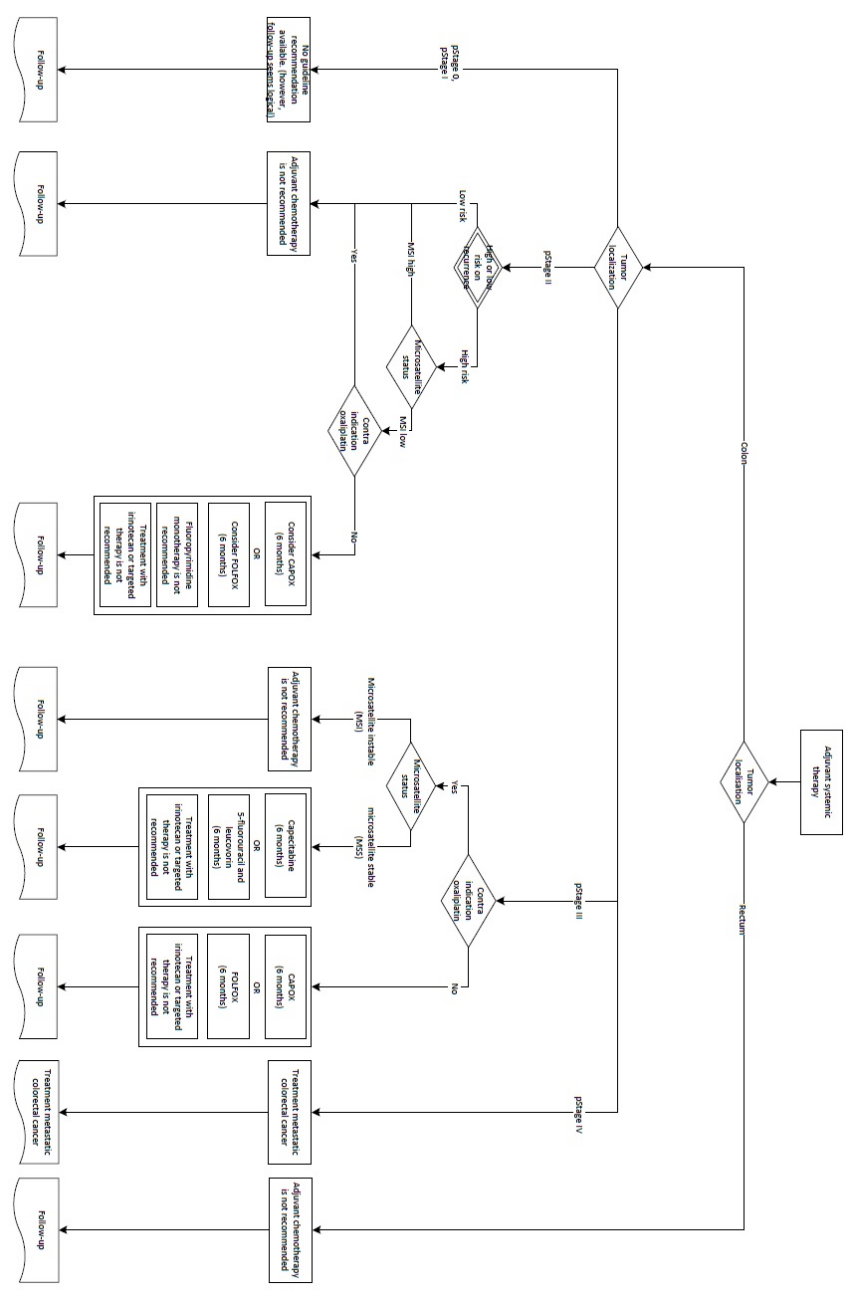
Decision tree to determine adjuvant treatment for colon cancer according to Dutch colorectal cancer guidelines.

**Figure 3.**
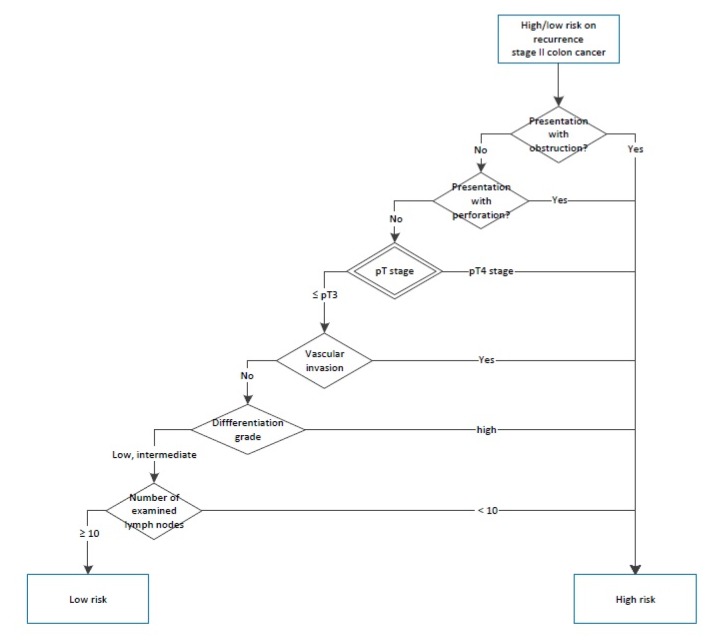
Decision tree to determine adjuvant treatment for colon cancer according to Dutch colorectal cancer guidelines.

### 2.2. Feasibility study

To assess the practicality of and illustrate the application of our proposed protocol, we performed a feasibility study using the Dutch colorectal cancer guidelines for the adjuvant setting. Following the protocol outlined in Part 1, we generated cases simulating patients that underwent resection of stage I-III colon cancer with curative intent and who might be eligible for ad¬juvant treatment with chemotherapy. We used this patient cat¬egory as a first example to evaluate concordance between the Dutch guidelines and Watson. We also chose for this patient cat¬egory as clear and straightforward guideline recommendations are available in the most recent Dutch guideline from 2014 [16] which facilitates comparison with Watson's treatment advice.

#### 2.2.1. Dutch colorectal cancer guidelines for the adjuvant setting of colon cancer

Adjuvant chemotherapy is not indicated for patients with stage I colon cancer because of their favourable prognosis. Adju¬vant chemotherapy may be considered for patients with high-risk stage II and is indicated for stage III colon cancer patients after resection of the primary tumor. High-risk stage II colon cancer is defined as having one or more of the following features: pT4 tumor, less than 10 examined regional lymph nodes, poorly or undifferentiated tumors, (extramural) vascular invasion, and/or presentation with obstruction/perforation. For these patients, adjuvant chemotherapy may be considered provided that their tumor is microsatellite stable (MSS). Patients with microsatel-lite instable (MSI) tumors and/or in whom oxaliplatin is con-traindicated should not be treated with adjuvant chemotherapy. For patients with stage III colon cancer, adjuvant chemotherapy consisting of a fluoropyrimidine (5-fluorouracil or capecitabine) with oxaliplatin is the regimen of choice. Patients with stage III colon cancer in whom oxaliplatin is contraindicated may be treated with fluoropyrimidine monotherapy as this still confers a survival benefit compared to observation alone, provided that the tumor is MSS. Adjuvant chemotherapy with fluoropyrimi-dine monotherapy is not indicated for stage III patients with MSI tumors.

#### 2.2.2. Generation of test cases and data collection

We established a minimum set of required variables to gener-ate synthetic patient cases. To do this, we used an adjuvant treat-ment decision tree from the Dutch guidelines (Figure 2 and 3) to identify all relevant variables, also called 'decision points' that led to a specific guideline recommendation. Decision points all together consisted of patient (e.g. age, functional status), clinical (e.g. contra-indication for oxaliplatin) and tumor characteristics (e.g. pT stage). Subsequently we developed an Excel file with one decision point per column and one synthetic patient case per row. Each synthetic patient had a unique combination of values for the decision point variables. We added 2 columns to enter Dutch guideline recommendations and Watson's treatment advice, which were subsequently analyzed using above described cross tabulations. The latest versions of the software were used (Oncoguide 1.1.0 and Watson 17.3).

#### 2.2.3. Additional analysis

In addition to following the protocol in section 2.1, we performed two additional analyses for our feasibility study: a comparison of the results to the US guidelines (to gain a sense of the degree to which non-concordance between Watson and Oncoguide is attributable to non-concordance between Dutch and US guidelines), and a usability assessment.

#### 2.2.4. Comparison to NCCN guidelines

We first compared the US National Comprehensive Cancer Network (NCCN) 2017 Clinical Practice guidelines [17] with the Dutch guidelines for adjuvant treatment of colon cancer (Table 4) to identify differences which might clarify why Watson's advice differed from Dutch guideline recommendations. Amer¬ican Society of Clinical Oncology (ASCO) guidelines for the treatment of colorectal cancer are not available, except for an outdated (2004) guideline regarding adjuvant chemotherapy for stage II colon cancer [18]. Next, we compared Watson's ad¬vice with the NCCN guideline recommendations using the same methods as the earlier prescribed in section 2.1.3 (Comparison with the Dutch guidelines).

#### 2.2.5. Usability

Another important aspect of evaluating a decision support system is evaluation of the system's usability. Serious usabil¬ity issues could lead to inability to use the system, or misin¬terpretation of the results. The interface offered for use in The Netherlands is a relatively simple form-based interface provided by MRDM. Patient data must be copied and entered into the form. As the primary goal of this evaluation was to determine whether this interface would be usable in subsequent testing, a cognitive walkthrough method was chosen [19,20]. Cognitive walkthrough is an evaluation performed by experts, in which a set of goals is specified along with the actions required to com¬plete the goals. The evaluator performs the actions, and at each step answers four questions:

1. Can the user identify the next step toward completing the task?

2. Can the user identify the action needed to complete that step?

3. Can the user correctly execute the action?

4. Can the user understand the feedback that the system gives after the action is taken?

As the current interface is fairly simple, all parts of the interface were evaluated. Additionally, we measured the data entry time for each case.

**Table 1.**
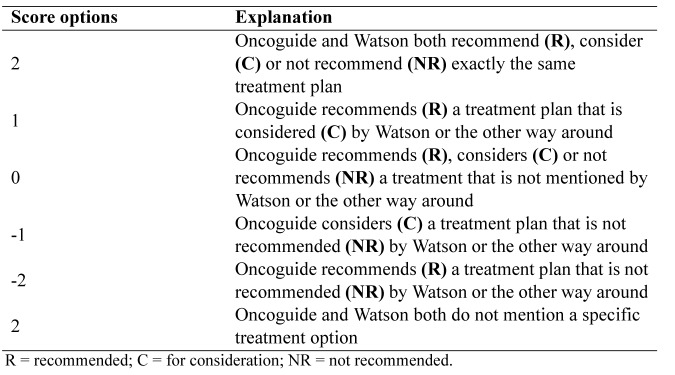
Overviewofanalysistable

## 3 Results

Our feasibility investigation resulted in an example of the results that can be expected by following the protocol outlined in section 2.1 of our methods, plus two additional analyses: a com-parison of the advice to the advice from US guidelines, and a usability assessment.

*Example results from protocol (Dutch colorectal cancer guidelines for the adjuvant setting): *We identified 9 decision points based on the Dutch guidelines and compared these vari¬ables with the minimum set of 13 variables (also decision points) required to obtain Watson's treatment advice (Table 5). Eight de¬cision points appeared in both sets, leaving a total of 14 unique decision points. Two decision points (less than 10 lymph nodes and (extramural) vascular invasion) from the Dutch guideline had to be modified to fit in the interface. Five decision points from Watson were not mentioned in the Dutch guideline and were therefore added to the synthetic cases. Of these, two (seri¬ous liver or kidney disease versus no serious comorbidity) were tested as variables in our feasibility study. The remaining three were held constant as normal/absent: resection margins and per-ineural invasion were entered as negative and functional status as zero (fully active, able to carry on all pre-disease functions without restriction).

In total, we developed 190 synthetic cases (stage I: 8 cases; stage II: 110 cases; stage III: 72 cases) and analyzed each case on treatment concordance. In Table 6, we present two examples of synthetic patient cases.

### 3.1. Comparison to Dutch guidelines

Overall concordance scores ranged between a minimum score of -4 (6 cases) to a maximum concordance score of +12 (17 cases) and concordance scores ranged per cancer stage (Fig¬ure 4). The median concordance score was +3. In total, 69 cases (36%) were labeled with red flags, 96 cases (51%) with orange flags and 25 cases (13%) without flags. Examples of analyzed cases are presented in Table 6.

### 3.2. Comparison to NCCN guidelines

Overall concordance scores ranged between a minimum score of —4 (9 cases) to a maximum concordance score of +12 (24 cases) and concordance scores varied per cancer stage (Fig¬ure 5). The median concordance score was +5. No orange or red flags were reported for the comparison between Watson and the NCCN guidelines.

**Table 2.**
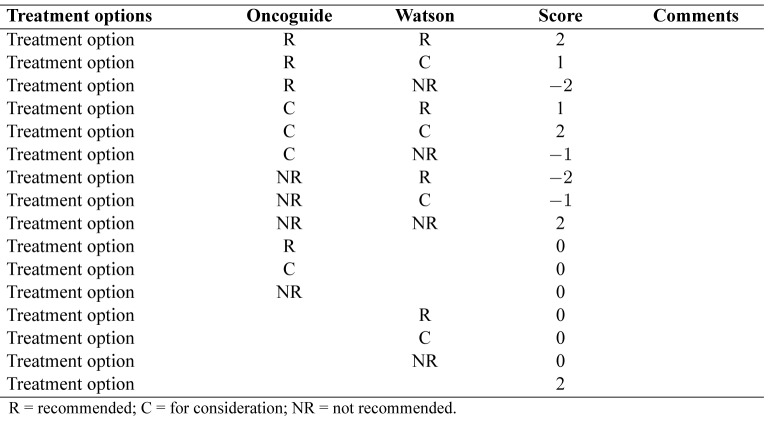
Overview of different score options.

**Table 3.**
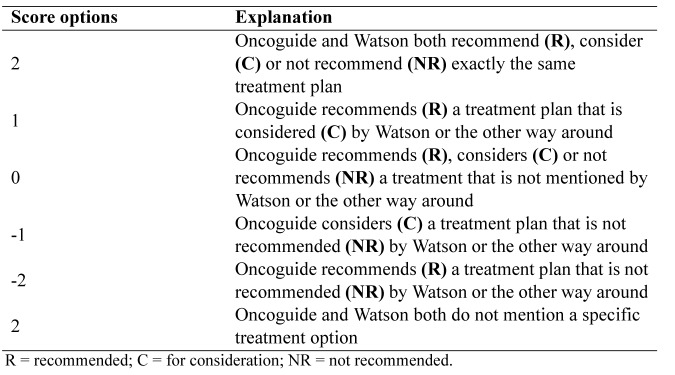
Explanation of different score options.

**Table 4.**
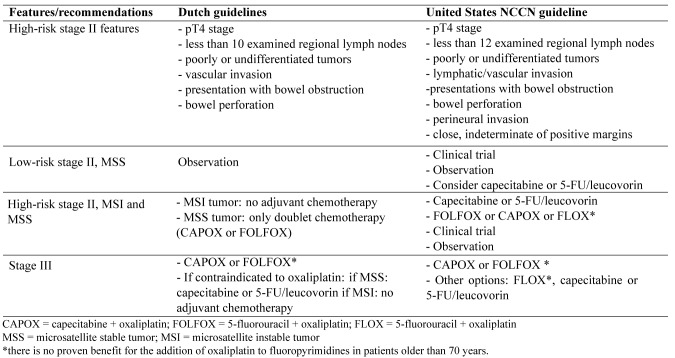
Overview of differences in guideline recommendations for colon cancer in the adjuvant setting between the Dutch and United States NCCN guidelines.

### 3.3. Usability

The users (LK, DvdB, and SZ) of the Watson interface were able to perform the tasks as described in the walkthrough method. Four steps were required for data entry. For each step, the users were able to identify the next step toward completing the task, identify the action needed to complete that step, cor-rectly execute the action and understand the feedback that the system gave after the action was taken. However, entering data in this way was subjectively perceived as cumbersome. Time spent on entering a single case was 10 minutes for the first case but quickly decreased to approximately 1.5 minutes per case af¬ter entering 20 cases (Figure 6). Watson generated treatment ad¬vice in a few seconds per case.

**Table 5.**
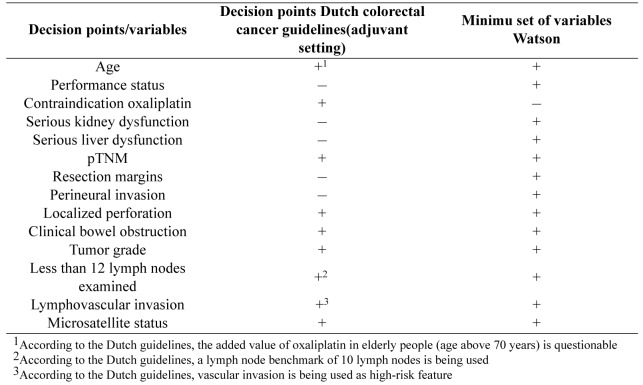
Overview of decision points based on the Dutch colorectal cancer guidelines and minimum set of variables of Watson for Oncology.

**Figure 4.**
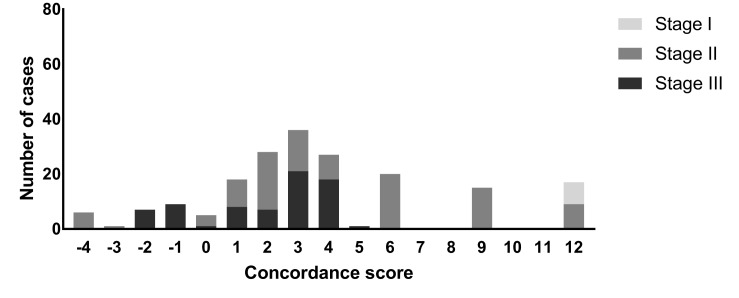
Concordance scores (Watson versus Oncoguide)differentiated by tumor stage.

**Figure 5.**
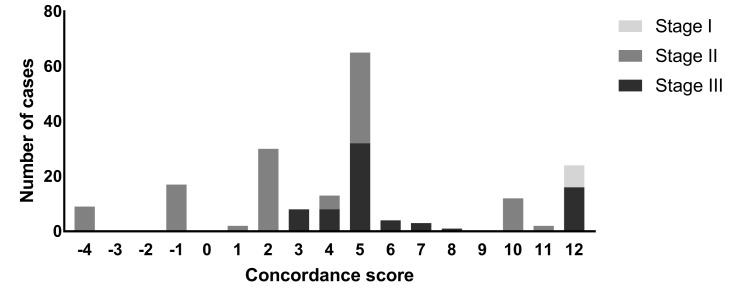
Concordance scores (Watson versus NCCN) differentiated by tumor stage.

## 4 Discussion

We successfully developed a protocol for the systematic evaluation of a black-box decision support tool. We used this protocol for an initial evaluation of Watson and concluded that the usability of Watson was acceptable, but concordance scores (for both comparisons between Watson and Oncoguide and Wat¬son and the NCCN guidelines) per case varied considerably. In our study, we identified several challenges that could contribute to further optimization and implementation of Watson in clinical practice.

Clinical decision support systems are typically evaluated in clinical trials, and evaluate whether the system changes the pro¬cess of care in ways which could affect clinical outcomes [21]. However, before determining whether advice is followed, it is first necessary to ensure that the system is providing the right advice. As outlined in the introduction, this is typically done by first comparing the system to the clinical knowledge on which it was based, then comparing the output of the system to the judg¬ment of clinical experts in a defined set of test cases. In Watson and other neural network systems, there is no specification to perform this first step. Thus, following the recommendations of Smith et al.[13], we have chosen to compare to another system. Oncoguide is intended to represent the standard of evidence-based care in The Netherlands (as it is a representation of the Dutch colorectal cancer guidelines in decision trees), thus it is a logical choice for evaluating the system for use in The Nether¬lands [22]. Comparison against an objective standard also adds value over only comparing with clinician judgment [23]. Given the large number of test cases we expect to generate, this also allows us to perform the evaluation more efficiently, as we can reasonably assume that if Watson and Oncoguide agree then the clinician will also agree. In many respects, a machine learning system can be viewed as a prediction model: the system "pre¬dicts" which treatment experts would recommend for this pa¬tient. Thus this evaluation can also be viewed as an "external validation" of this model: US-based experts trained the system, and it is not known if its recommendations will be valid in an-other setting.

This protocol introduces two new methods for evaluating ar¬tificial intelligence-based decision support systems: a method for generating synthetic cases, and the scoring system for as¬sessing agreement. Synthetic cases are generated based on the known inputs and outputs of the comparison system (in our ex¬ample, Oncoguide), and input from clinical experts on variables, which might indicate a justifiable departure from the guideline. This approach should capture both cases where Watson is likely to agree with the guideline, and cases where Watson may be able to offer better advice than the guideline. As with other decision support systems, simple "agreement" is not sufficient to describe the performance of this system [8]. Friedman and Wyatt pro¬posed the use of contingency tables when evaluating decision support systems, to make explicit the difference between false positive and false negative classifications. Their reasoning is that a false-positive error, such as erroneously suggesting a di¬agnosis for a healthy patient, may be less serious than a false-negative error, which may in turn be less serious than proposing the wrong diagnosis entirely [8]. Likewise, a suggestion from the system that the clinician "consider" a treatment, which is in fact contraindicated, is a less serious error than "recommend¬ing" use of that treatment. Thus, we have extended the notion of a contingency table to express the idea that some disagreements have greater consequences than others.

Preliminary results from other concordance studies with Watson appear in the literature, primarily in conference proceed¬ings. Oncologists at Bumrungrad International Hospital in Thai¬land compared Watson's recommendations to the recommenda¬tions of their own oncologists [10]. They found 89% concor¬dance for colorectal cancer patients, which is substantially better than concordance levels in our feasibility study. An earlier study in India reported 81.0% concordance for colon and 92.7% con¬cordance for rectal cancer [12]. A study in South Korea found 85% concordance for colon cancer in the adjuvant setting [24]. Although thus far these studies have only been reported in ab¬stract format, the reported methods suggest three possible rea¬sons for the differences. First, these studies used real patients, which (as discussed below) is a different outcome than in our study. Second, they used a simple definition of concordance: if the treatment selected by oncologists appeared in either the 'recommended' or 'for consideration' part of Watson's advice, then the recommendation was considered concordant. By con-trast, our approach allows for different levels of disagreement: if Watson recommends a treatment that is contraindicated by the Dutch or NCCN guideline, that is a more substantial disagree¬ment than simply not mentioning a treatment recommended by our guideline, or suggesting a treatment "for consideration" that the Dutch or NCCN guideline omits. Our approach captures and quantifies this difference. Finally, the guidelines used in these countries may more closely parallel US guidelines. In the Ko¬rean study it was noted that most of the observed disagreements were attributable to differences with the US guidelines.

We performed an additional comparison of Watson versus the NCCN guidelines to gain a sense of the degree to which non-concordance between Watson and Oncoguide was attributable to non-concordance between Dutch and US guidelines. We iden¬tified variety in the concordance scores in both situations, but no orange or red flags were reported for the comparison be¬tween Watson and the NCCN guidelines. This supports that dis¬agreements between Watson and Oncoguide are (partially) at¬tributable to guideline differences.

We chose to use synthetic cases rather than real cases to per¬form our evaluation, because our main goal was to detect differ¬ences between Watson's advice and our local guidelines. This means that our evaluation did not measure how often Watson's advice would agree with the guideline in practice and real pa¬tient cases. We attempted to create a heterogeneous dataset of synthetic patient cases to overcome this issue, but our dataset was still limited in terms of variation (e.g. age was restricted to 3 levels: 45, 60 and 75 years and kidney or liver diseases were present or absent). This was due the need to manually enter all cases. An easier way to import a larger dataset of patient cases is by using an application-programming interface (API), but this option was unfortunately not available in the timeframe of our study. If an API were to be provided for automated entry of (test) patient data, then we could exhaustively test of all possible com¬binations of variables, including continuous variables (e.g. per year for age and different levels of kidney and/or liver disease). We intend to repeat out study with patients with more compli¬cated features.

We elected to use a simple, expert-based usability evalua-tion, the cognitive walkthrough approach. We considered this approach to be appropriate to the circumstances of this evalu-ation: the tasks to be accomplished in the system were well-defined, and the main goal was to identify usability issues that could be a barrier to use of the system in a trial setting with naive users [20]. Furthermore, the interface itself is relatively simple, and more qualitative methods such as think-aloud were, in the authors' view, unlikely to yield additional insights. Although no usability problems were identified and the system is usable for our proposed evaluation, the workflow of hand-entering data is cumbersome. Direct interoperability with a patient record database would be preferable, but it is neither available for the electronic health record in use at our hospital nor for synthetic cases.

In our study, we calculated overall concordance scores per case between Watson's advice and the Dutch guidelines and used orange and red flags to indicate differences in treatment recom¬mendations. However, we did not decide whether discordance is actually a positive or negative change regarding to the orig¬inal Dutch guideline recommendation. In other words, we did not conclude whether Watson's advice may be actually better or worse than the Dutch guideline recommendations. Future re¬search should therefore critically evaluate each case of discor¬dance, including evaluation by oncologists of the clinical sound¬ness of the recommendation and examining the literature refer¬ences Watson provides to see if they justify its recommendation. Future work could also include an assessment of whether the lit¬erature that Watson provides could in and of itself be of value to the decision-making process.

Watson's approach of generating personalized treatment ad¬vice by integrating natural language processing with evidence from guidelines and studies, case-based reasoning, and machine learning based on training by expert oncologists is definitely promising in terms of revolutionizing daily clinical practice with the ongoing expansion of medical literature. Watson also has the potential to conquer two difficult problems from decision support perspective, namely that of poorly structured medical record data and maintaining the knowledge base. However, if further implementation of Watson outside the United States is being pursued, future research should focus on further evaluation after localization of Watson with adjustments based on national or local guidelines. "Black-box" systems such as Watson im¬pose a risk that other decision support systems do not, in that we cannot know exactly how the system arrives at its conclusions. For example, Oncoguide does not consider the patient s age in its recommendations; its users are aware of this and compensate accordingly. Watson may or may not be considering age, and its end users have no way to know when it does, or whether this may change with a new version update. A partial solution could be to maintain a suite of test patients as we ve proposed in our protocol, and to run these tests regularly. Then clinicians could be made aware if its recommendations change for some groups of patients.

In conclusion, a systematic evaluation of a "black-box" deci¬sion support tool is feasible using synthetic patient cases and em¬pirically testing the outcomes. Several reasons for discordance of a decision tool with synthetic cases should be considered, but disagreements were undoubtedly partially attributable to differ¬ences between the Dutch and US guidelines. This may imply that Watson needs to be re-trained by local experts to reflect dif¬ferences in the local care setting. Localization of a cognitive decision support tool (e.g. Watson) based on local guidelines is therefore essential before considering further external validity studies and implementation in daily practice.

**Table 6.**
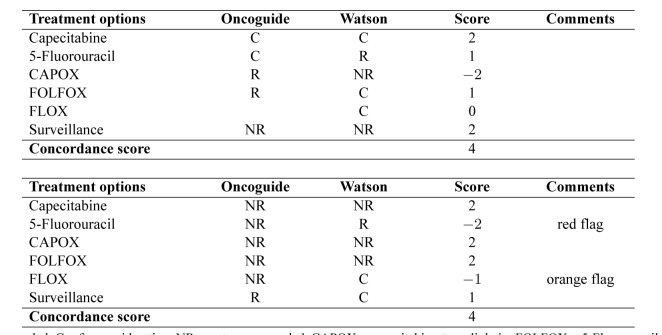
Examples of two analyzed cases of colorectal cancer patients in the adjuvant setting.

**Figure 6.**
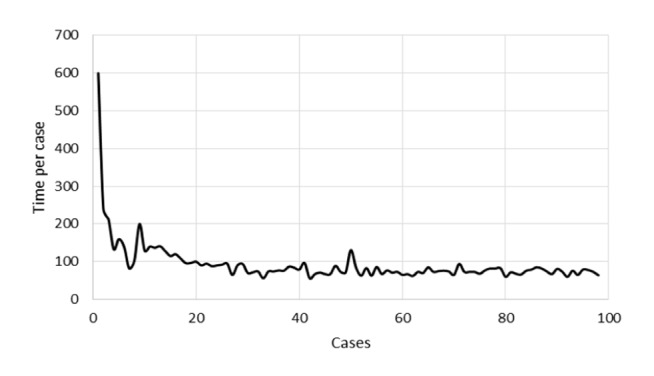
Entrytimepercase.
